# Cryosphere and Psychrophiles: Insights into a Cold Origin of Life?

**DOI:** 10.3390/life7020025

**Published:** 2017-06-11

**Authors:** Georges Feller

**Affiliations:** Center for Protein Engineering–InBioS, Institute of Chemistry B6a, University of Liège, B-4000 Liège-Sart Tilman, Belgium; gfeller@ulg.ac.be; Tel.: +32-4-366-33-43

**Keywords:** cryosphere, psychrophiles, ice, eutectic phase, RNA world, ribozyme

## Abstract

Psychrophiles thrive permanently in the various cold environments on Earth. Their unsuspected ability to remain metabolically active in the most extreme low temperature conditions provides insights into a possible cold step in the origin of life. More specifically, metabolically active psychrophilic bacteria have been observed at −20 °C in the ice eutectic phase (i.e., the liquid veins between sea ice crystals). In the context of the RNA world hypothesis, this ice eutectic phase would have provided stability to the RNA molecules and confinement of the molecules in order to react and replicate. This aspect has been convincingly tested by laboratory experiments.

## 1. Introduction

The oldest traces of potentially biogenic carbon on Earth have been recently dated back to 4.1 billion years [1], while the most ancient microfossil records have been found in the range of 3.2 to 3.7 billion years [2,3,4]. These cells were all expected to thrive in shallow marine costal environments, which suggests mild local temperatures. Fossil records from the most primitive cellular organisms remain elusive because their soft structure has only left very discrete chemical signatures, and also because of the lack of rock records that old. Nevertheless, hyperthermophilic prokaryotes (mainly archaea) have long been regarded as possible remnants of these primitive living forms [5,6] because they branch deep in the phylogenic tree of life, with short branches towards the last universal common ancestor (LUCA), even though the structure of the tree remains under debate [7,8] with recent evidence for a two-domain tree of life [9]. Furthermore, only extant archaeal species are able to colonize the most extreme environments on our planet in terms of temperature, pH, or salinity [10]. However, this view seems to be tainted by an anthropocentric bias because it postulates that extant hyperthermophilic prokaryotes would have escaped about 3.8 billion years of evolution, which is more than suspicious. As a matter of fact, current views suggest that LUCA was a mesophilic organism and that extant extremophiles have subsequently colonized harsh environments [11,12,13,14,15,16]. In this respect, the occurrence of *sn*2,3 di- and tetra-isoprenoid ethers in archaeal membranes (instead of *sn*1,2 di-fatty acyl esters in the other domains) appears as a key determinant of archaea to outperform in extreme environments [15]. As far as prebiotic chemistry is concerned, deep sea hydrothermal vents have been proposed to be a plausible natural phenomenon compatible with the synthesis of the first biogenic molecules [17]. These hot vents currently support life under extreme conditions, their chemistry agrees with a chemolithoautotrophic primitive metabolism [18], and their isolation in deep sea water would have protected from intense UV radiation detrimental to biological macromolecules [19].

Although attractive and well documented [20], the hypothesis of hot hydrothermal vents is in partial contradiction with the concept of the RNA world [21,22], which postulates that RNA was the first biogenic macromolecule because it is endowed with catalytic activity (the ribozyme) and genetic information, in contrast with DNA and proteins. The contradiction mainly resides in the pronounced heat-lability of the isolated RNA molecule, and has led to the proposal of a cold origin of life [23]. Furthermore, the mechanism of enzymatic activity requires some proximity with the substrates, which is not met in the concept of a primitive soup. Accordingly, the possibility that ice has been a matrix for biogenic molecules has emerged. In this respect, extant psychrophiles have never been consistently regarded as remnants of a cold origin of life, but their ability to colonize the most extremely cold environments provides physicochemical evidences supporting the appearance of biotic macromolecules in the cold, which should not be neglected amongst the various hypotheses [24,25].

## 2. Extant Psychrophiles

It is frequently overlooked that the majority (>80%) of the Earth’s biosphere is cold and permanently exposed to temperatures below 5 °C [26]. Such low mean temperatures mainly arise from the fact that ~70% of the Earth’s surface is covered by oceans that have a constant temperature of 2–4 °C below 1000 m depth, irrespective of the latitude. The polar regions account for another 15%, to which the glacier and alpine regions must be added, as well as the permafrost representing more than 20% of terrestrial soils. All these low temperature biotopes have been successfully colonized by cold-adapted organisms (termed psychrophiles [27]), which include a large range of representatives from all three domains—Bacteria, Archaea, and Eukarya. These organisms do not merely endure such cold and inhospitable conditions, but are irreversibly adapted to these environments, as most psychrophiles are unable to grow at mild (or mesophilic) temperatures. Some microorganisms have a larger growth temperature range, from low to mild temperatures, sometimes referred to as psychrotrophs or psychrotolerants. However, in the absence of environmental, physico-chemical, or metabolic parameters to differentiate them, any organism thriving and dividing actively at low temperature is currently defined as a psychrophile [28,29].

Psychrophiles thrive in permanently cold environments in thermal equilibrium with the medium and even at sub-zero temperatures in supercooled liquid water. Such extremely cold conditions are encountered, for instance, in salty cryopegs at −10 °C in the Arctic permafrost [30], in the brine veins between polar sea ice crystals at −20 °C [31,32], or in supercooled cloud droplets [33]. The bacterium *Planococcus halocryophilus*, isolated from high Arctic permafrost, was found to divide at −15 °C and to remain metabolically active at −25 °C [34], which probably represents the lower temperature limit before dormancy. Unusual microbiotopes have also been described, such as porous rocks in Antarctic dry valleys hosting microbial communities surviving at −60 °C [35]. Cryoconite holes on glacier surfaces represent another permanently cold biotope hosting complex microbial communities [36]. Antarctic fungi and cryptoendolytic communities found, for instance, in Antarctic Dry Valleys are regarded as the most psychrophilic eukaryotes [37], while polar fish thriving at −2 °C beneath the icepack are the biggest psychrophiles [38]. Glacier ice worms are also worth mentioning, as they complete their life cycle exclusively in glacier ice [39]. These examples highlight the unsuspected ability of psychrophiles to adapt to low temperatures. Evidently, life in cold environments requires a vast array of adaptive features at nearly all levels of the cell architecture and functions [29,40,41,42].

There is no formal lower temperature limit for life under natural conditions, as most microbial species can be maintained for extended periods of time in low temperature freezers at −80 °C, then revived under appropriate conditions. Bacterial survival has been reported in frozen permafrost samples up to half a million years in age, and such viability was correlated with the capacity to slowly repair DNA, therefore preserving the cell genetic program [43].

## 3. Life in the Cryosphere

The concept of ice as a habitat for cold-adapted organisms has been initially described by Priscu and Christner [44]. Currently, this field is intensively investigated, and is the topic of international conferences [45]. The microbial ecology of the cryosphere has benefited from the most recent omics approaches [46,47,48,49,50]. More specifically, Price has provided a series of significant contributions focused on physico-chemical and energetic aspects of life in ice [51,52,53,54,55], summarized in [56]. The above-mentioned reports, and especially [52,56] should be consulted for a complete coverage of the topic.

In the context of a possible cold origin of life, some parameters are worth mentioning here, as they are relevant for both extant psychrophiles and prebiotic macromolecules.

### 3.1. Microenvironments in Ice

Three habitats for psychrophiles in ice have been described.

**Figure 1:**
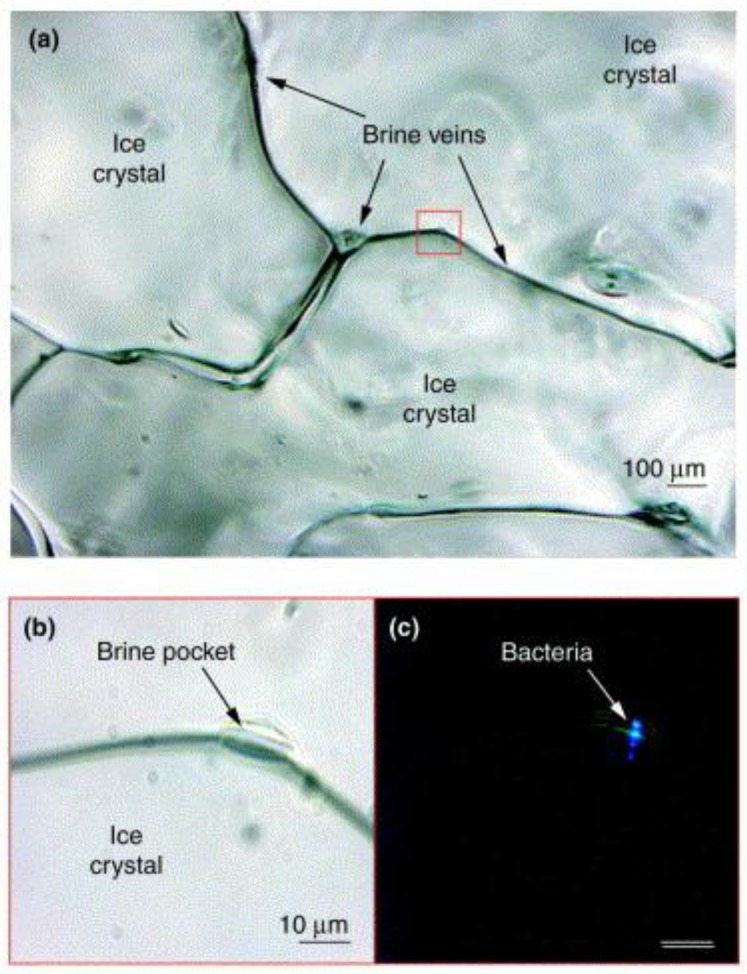
Bacteria visualized microscopically directly within a brine pocket of Arctic winter sea ice at −15 °C: (**a**) The transmitted light image shows ice crystals and the brine-filled veins between them; (**b**) Enlarged image of a brine pocket in (a) shows the microscale habitat; (**c**) Its bacterial inhabitants are revealed by epifluorescence microscopy [31], reprinted by permission from Elsevier.

(i) Liquid veins between ice crystals: the eutectic phase [51]. In glacier ice, solutes are excluded from ice crystals and are concentrated in interstitial liquid veins. In sea ice, the concentrated sea salts allows these brine veins to remain liquid to −35 °C [31] and to host microorganisms in a network of micron-diameter veins, as illustrated in Figure 1.
(ii) Unfrozen water film in contact with minerals. Ice in contact with rocks, mineral particles, or clay grains harbor a nanometer-thick layer of unfrozen water. Cells attached to mineral particles have been observed in both glacier ice and permafrost. As a result of the small size of the water film, these cells are immobilized in this microenvironment and are not able to move freely.
(iii) Inside ice crystals. Microorganisms can be trapped inside individual crystals in polycrystalline ice [54]. As with mineral inclusions, a microbe will be coated with an unfrozen layer of water. Interestingly, such a microenvironment is potentially less detrimental to cells than veins and minerals which possess a hostile chemical composition.

It follows that ice, but also frozen minerals or clay and permafrost, all display liquid microenvironments, ranging from thin water molecule layers to large veins, which are compatible with the water-based chemistry of prebiotic molecules.

### 3.2. In-Ice Metabolism

Cells can remain metabolically active in the above-mentioned microenvironments, as they can obtain a limited amount of gases and nutrients by diffusion, including through individual ice crystals, whereas waste products diffuse away from them. For instance, huge excess concentrations of CO_2_ and CH_4_ in deep Greenland glacier ice have been related to ongoing *in situ* production by microorganisms trapped in ice 400,000 years ago [55,57].

**Figure 2:**
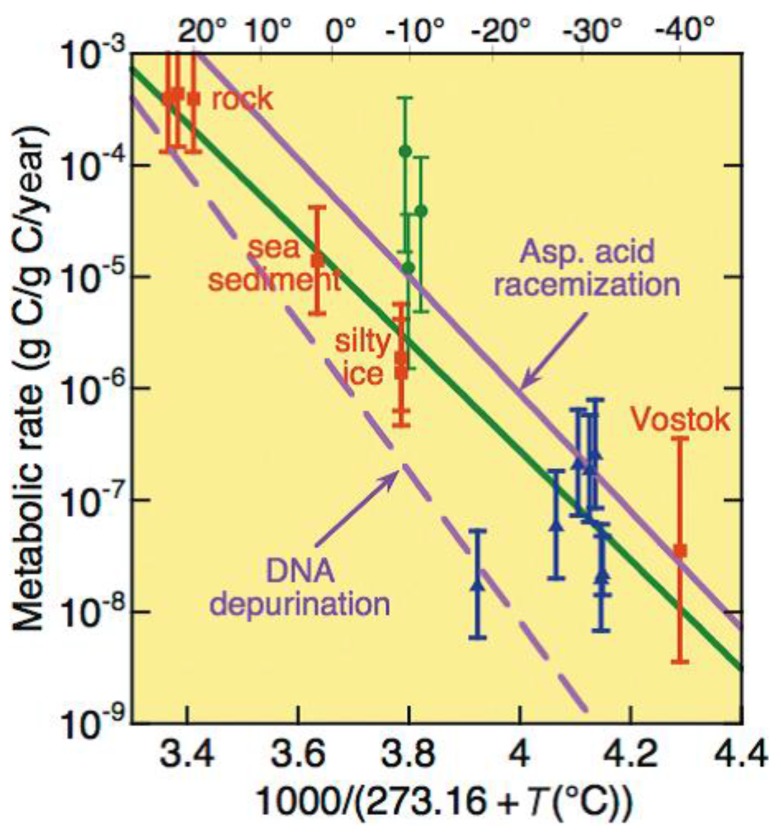
Metabolic rate for fractional turnover of carbon in cells trapped in ice, rock, and sediment as a function of in situ inverse absolute temperature (upper scale in °C). The purple lines are extrapolated from rates of racemization of aspartic acid and of DNA depurination. The green solid line is an exponential fit to the metabolic data [55,56], reprinted by permission from Canadian Science Publishing.

Exponential growth with doubling time in the range of 5 h at 0 °C recorded for psychrophilic bacteria cultivated in the laboratory in rich medium [58] are unlikely to occur under environmental conditions. Nevertheless, seasonal blooms of algae attached beneath polar ice sheets are very fast and productive events, stimulated by summer light crossing the ice cover [59]. A maintenance metabolism ensuring the basal cell functions but without growth is expected for airborne microorganisms deposited on snow and ice, or for marine microorganisms entrapped in seasonal polar sea ice, if they are not adapted. By contrast, microbes imprisoned in ice for extended periods of time enter in a survival metabolism (or dormancy) without division in which the limited amount of available energy is devoted to the repair of macromolecular damages [53]. Figure 2 illustrates an Arrhenius plot of metabolic activity as a function of temperature down to −40 °C (upper scale). The metabolic rate is given in g carbon (of metabolic gas) per g carbon (of cellular origin) per year [55]. There is no obvious break in this plot: the metabolic activity exponentially decreases with low temperatures, just like any elementary chemical reaction. Furthermore, the recorded metabolic activities are clustered with the rates of nucleic acid depurination and amino acid racemization: the low residual metabolic activity is therefore just sufficient to repair these spontaneous macromolecular damages, as also evidenced by the most ancient bacteria recovered from ice [43].

In the context of prebiotic chemistry, the main message here is that extremely low environmental temperatures do not totally abolish the metabolism of a complex microorganism like a bacterium, which relies on a huge number of biochemical reactions. Accordingly, simple prebiotic chemical reactions might have been slowed down but not cancelled in ice at low temperature.

### 3.3. In-Ice Survival

Vitrification of the intracellular content—which does not result in cell death (in contrast to freezing)—has been proposed for survival at very low subzero temperature [60]. However, the limit for survival in ice is apparently related to the exhaustion of energy supply, which leads to cell death. Besides macromolecular aging, natural radiation has also been implicated in cell decay. Mineral inclusions in ice and permafrost contain natural uranium and thorium isotopes, the decay of which generates α particles. Such radiation induces DNA single- or double-strand breaks and produces reactive oxygen species. In addition, the time-dependent DNA degradation in the oldest ices from Antarctica has been related to the generation of ionizing radiation from cosmic high-energy particles [61].

The long term survival of microorganisms in ice suggests that diffusion of nutrients and waste products is still effective in frozen environments. It follows that prebiotic reactions might have been confined in an icy medium, but with sufficient diffusion potential to avoid poisoning of the niche. Furthermore, ice appears as a better environment to escape natural radiation as compared with minerals and permafrost, although none of these environments could protect from cosmic radiation until buried several meters in ice.

## 4. Ice as a Protocellular Medium for Life

Several studies have shown that the polymerization of prebiotic molecules is favored in ice veins and at the surface of clay grains at subfreezing temperatures because precursors are concentrated from initially dilute solutions and are organized, for instance, on a mineral surface that provides a scaffolding, while low temperature reduces the tendency to hydrolyze (see [52,62,63] for a discussion). However, in the context of the RNA world hypothesis, an additional constraint is imposed because these molecules have to replicate. 

**Figure 3:**
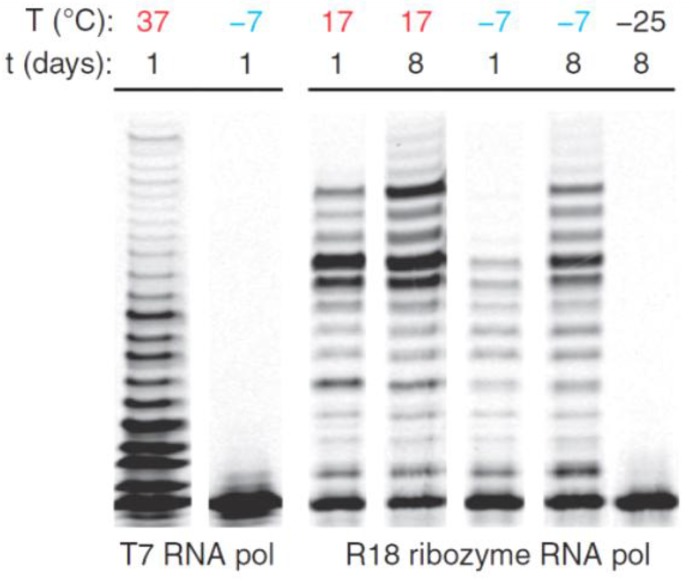
Fluorescent primer extension reactions using a proteinaceous polymerase (T7 RNA polymerase) and the ribozyme RNA polymerase at ambient temperatures (red) and in ice (blue) resolved by gel electrophoresis [64], reprinted by permission from Macmillan Publishers Ltd.

In this respect, an outstanding contribution in this field from the Holliger’s lab has convincingly shown that ice can be a protocellular medium for RNA replication [64]. The authors used a ribozyme to monitor in-ice RNA-catalyzed RNA replication within the eutectic phase. They found that ice promotes high-fidelity RNA replication and protects the ribozyme from hydrolytic degradation, enabling the synthesis of long replication products for extended periods of time at −7 °C. This is in sharp contrast with proteinaceous RNA polymerases, which are inactivated by freezing (Figure 3). 

It was also shown that the eutectic phase is required for polymerase activity, as further freezing to −25 °C abolished ribozyme activity (Figure 3). Furthermore, highly dilute solutions of nucleotides and ions at concentrations found in present freshwater sources strongly impair ribozyme activity, whereas after freezing, a >200-fold concentration in ice veins promotes a near-optimal RNA replication activity. Finally, the eutectic phase slows down ribozyme diffusion by more than three orders of magnitude, providing quasicellular compartmentalization of RNA replication.

However, the ribozyme used in this study was optimally active at ambient temperature, and only synthesized short nucleotides. Therefore, the same group has subjected the ribozyme to in-ice directed evolution and engineering in order to produce a variant adapted to subzero temperatures able to synthesize RNA in ice at temperature as low as −19 °C [65]. This reveals that mutations in the RNA sequence are able to confer adaptive traits to the eutectic phase conditions. Furthermore, this ribozyme variant was capable of catalyzing the accurate synthesis of RNA sequences longer than itself—an important step toward RNA-self replication. It was also shown that freeze–thaw cycles promote the assembly of short RNA oligomers into an active ribozyme [66]. This outlines how cyclic physicochemical processes could have driven an expansion of RNA compositional and phenotypic complexity from simple oligomer pools in ice.

## 5. Conclusions

It is currently elusive to draw a precise scenario for the emergence of life, which should obviously include a wide range of diverse factors such as geothermal chemistry, meteoric input of organic precursors, clay geochemistry, or vesicles formation, amongst others [24,25]. Nevertheless, current evidence indicates that life appeared on Earth much earlier than previously thought—possibly before 4.1 billion years [1]. Within this timespan, the young Earth has presumably been subjected to several freezing periods [67]. Our anthropocentric view has neglected or refuted the possible appearance of life in icy environments, but extant psychrophiles demonstrate that life persists in the present coldest conditions [52,56]. Direct observation and laboratory experiments have revealed that liquid water films or veins occur in many icy microenvironments, compatible with water-based prebiotic and biotic chemistry. Furthermore, freezing concentrates precursor molecules in the ice eutectic phase to levels compatible with the polymerization of prebiotic molecules [62] and even with RNA self-replication in the context of the RNA world hypothesis [64], while low temperature should have protected these molecules from thermal degradation [23]. Such concentration and diffusion-limited conditions also provided quasicellular compartmentalization [64], which might have promoted the appearance of LUCA.

Ice is certainly not the unique environment for the origin of life, but as elegantly stressed by Price [56], “It is fascinating to note that the physico-chemical properties of clay grains and liquid veins in ice seem to play similar roles both in enhancing the rate of polymerization of biomolecules in Earth’s early history and in providing habitats for microbial life in modern glacial ice”.
